# Biolayer interferometry for DNA-protein interactions

**DOI:** 10.1371/journal.pone.0263322

**Published:** 2022-02-02

**Authors:** John K. Barrows, Michael W. Van Dyke

**Affiliations:** Department of Chemistry and Biochemistry, Kennesaw State University, Kennesaw, GA, United States of America; Consiglio Nazionale delle Ricerche, ITALY

## Abstract

Biolayer interferometry (BLI) is a widely utilized technique for determining macromolecular interaction dynamics in real time. Using changes in the interference pattern of white light reflected off a biosensor tip, BLI can determine binding parameters for protein-protein (*e*.*g*., antibody-substrate kinetics) or protein-small molecule (*e*.*g*., drug discovery) interactions. However, a less-appreciated application for BLI analysis is DNA-protein interactions. DNA-binding proteins play an immense role in cellular biology, controlling critical processes including transcription, DNA replication, and DNA repair. Understanding how proteins interact with DNA often provides important insight into their biological function, and novel technologies to assay DNA-protein interactions are of broad interest. Currently, a detailed protocol utilizing BLI for DNA-protein interactions is lacking. In the following protocol, we describe the use of BLI and biotinylated-DNA probes to determine the binding kinetics of a transcription factor to a specific DNA sequence. The experimental steps include the generation of biotinylated-DNA probes, the execution of the BLI experiment, and data analysis by scientific graphing and statistical software (*e*.*g*., GraphPad Prism). Although the example experiment used throughout this protocol involves a prokaryotic transcription factor, this technique can be easily translated to any DNA-binding protein. Pitfalls and potential solutions for investigating DNA-binding proteins by BLI are also presented.

## Introduction

Many biochemical studies revolve around understanding the interactions between macromolecules and other molecules. Examples include protein-protein interactions, protein-drug interactions, and nucleic acid-protein interactions. Numerous binding assays currently exist to study these molecular interactions, and new technologies and methodologies are constantly being explored [1]. Biolayer interferometry (BLI) is an experimental technique that determines interaction kinetics between two or more molecules of interest [2]. BLI analyzes the difference in interference patterns of white light reflected off a reference layer and biolayer. The biolayer is conjugated to a molecule of interest and then introduced into a solution containing other molecules of interest. Interactions between the free and stationary molecules alter the interference pattern, leading to a change in optical wavelength that is recorded in real-time.

Although BLI is widely accepted as a suitable binding assay for a variety of macromolecules, most BLI experiments involve protein-protein or protein-small molecule interactions [3–5]. Conversely, DNA-protein interactions are less commonly assayed by BLI, with few results published to date [6–8]. Understanding DNA-protein interactions is a cornerstone for several fields of study, including biochemistry, molecular biology, and medicinal chemistry. Additionally, with the advent of computational biology, the ability to predict DNA-binding sequences from a protein’s primary structure provides a powerful predictive tool for scientists investigating potential DNA-protein interactions [9–11]. As such, the use of experimental techniques to validate these findings is paramount.

BLI presents several advantages over traditional DNA-protein binding assays. Notably, the automated nature of this technique reduces human error. Additionally, many previous assays, such as DNase I footprinting, restriction endonuclease protection assay (REPA), or electrophoretic mobility shift assay (EMSA), cannot provide binding kinetics in real-time. Several established assays require fluorescent or radio-labeled substrates, while BLI can be used with unlabeled protein molecules and biotinylated-DNA, which is readily available from commercial vendors. Additionally, BLI does not require a secondary capture-based step, a major advantage when studying proteins that lack commercially available antibodies.

Here, we demonstrate the practicality of assaying DNA-protein interactions by BLI. We first use PCR to generate biotinylated-DNA substrates. Using the Octet RED96e system, these DNAs are probed against varying concentrations of a protein of interest. Changes in optical wavelength are measured in real-time, and these data are then used to ascertain association and dissociation rates using GraphPad Prism software.

## Materials and methods

This protocol described in this peer-reviewed article is published on protocols.io, https://dx.doi.org/10.17504/protocols.io.bx9spr6e and is included for printing as S1 File with this article.

## Expected results

**Fig 1:**
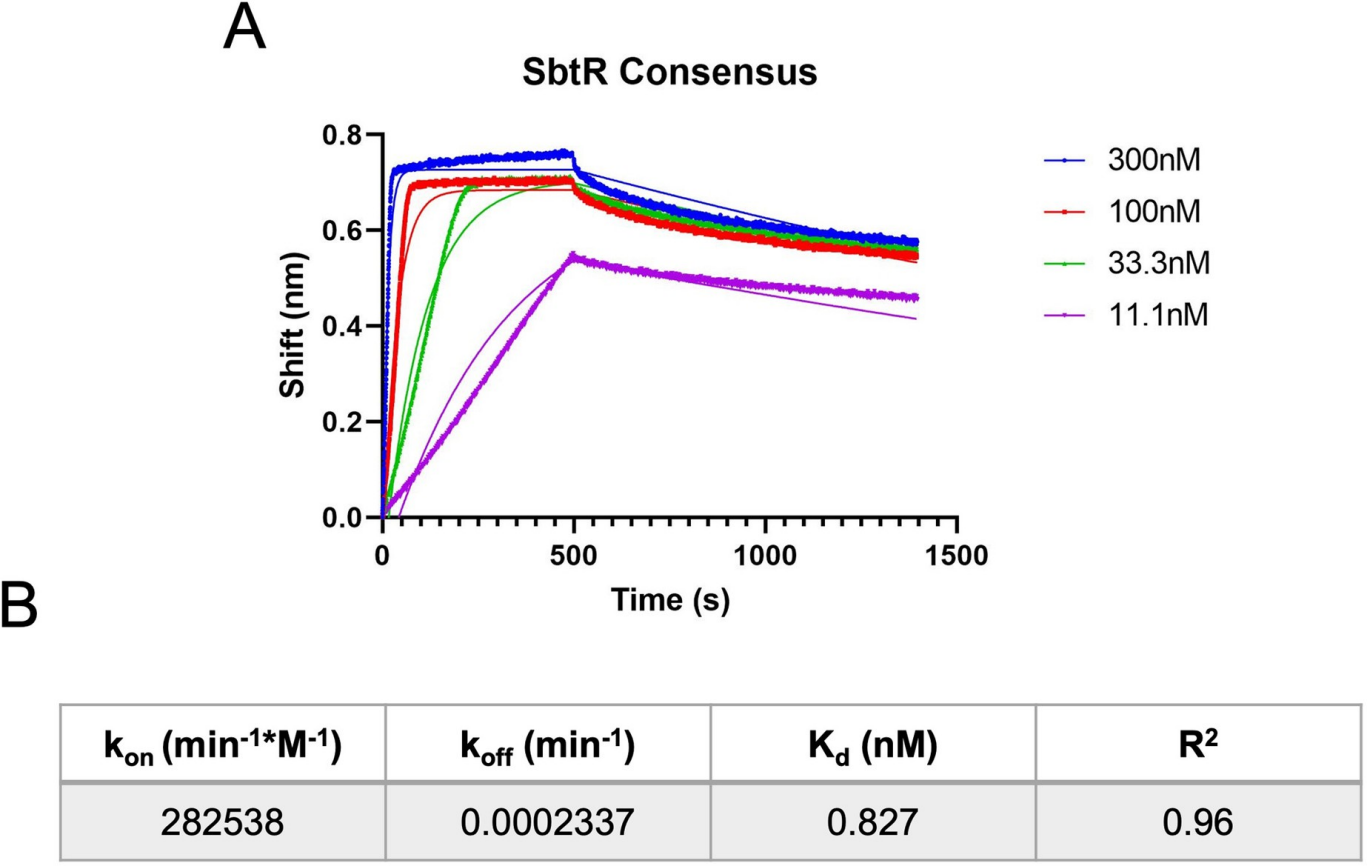
Association and dissociation of SbtR to its consensus DNA-binding sequence. (**A**) Various concentrations of SbtR were subject to BLI analysis. BLI was performed at 30°C in BLI-100 buffer (20 mM Tris-Cl [pH 7.5], 100 mM NaCl, 1 mM EDTA, and 0.05% Tween-20). Prior to the association step, streptavidin-coated biosensors were conjugated to biotinylated DNA probes containing a previously identified consensus DNA-binding motif for SbtR. In the graph shown, association occurred during the first 500 seconds, then samples were transferred to a buffer-containing well to measure dissociation. Solid lines depict lines of best fit from GraphPad Prism software. Dots represent individual BLI data points, that were taken every 0.2 seconds. (**B**) Kinetic data derived from the experiment shown in (A) are presented.

**Fig 2:**
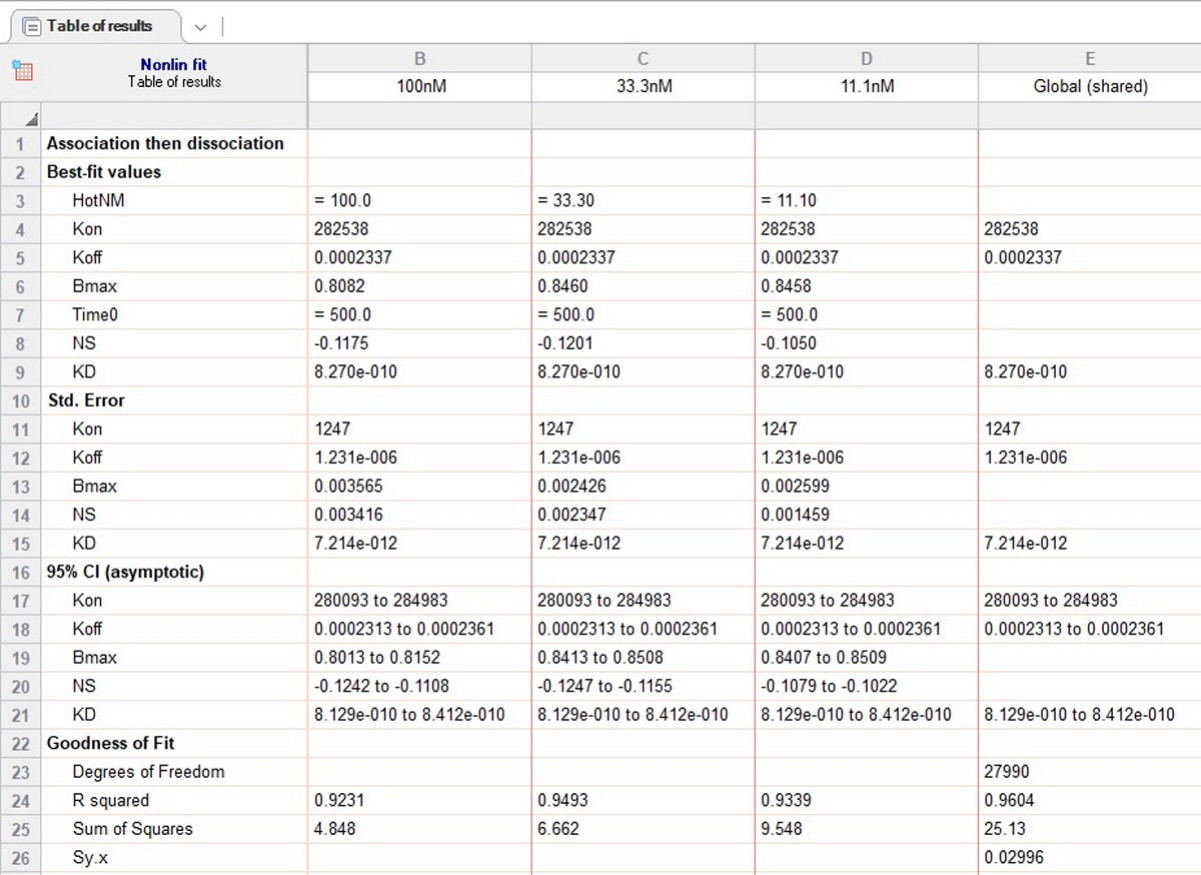
GraphPad Prism data output. A screenshot of results from the GraphPad Prism “Association then Dissociation” model for SbtR binding to its consensus sequence is presented.

By following the referenced protocol (also available in S1 File), one should be able to attain association and dissociation rates for a protein of interest to specific DNA sequences. Following the experimental example present throughout the protocol, we obtained data regarding a change in interference during which four different concentrations of SbtR were associating with the derived SbtR-DNA consensus sequence, followed by dissociation of the SbtR-DNA complexes upon >300-fold dilution (Fig 1A). Following analysis using GraphPad Prism software and their *Association then Dissociation* equation, the best-fit curves yielded global binding kinetics of *k*_on_ = 282538 min^-1^*M^-1^, *k*_off_ = 0.0002337 min^-1^, and a *K*_d_ of 0.827 nM (Figs 1B and 2). Statistical significance of these values, including standard errors, 95% confidence intervals, and goodness of fit (R^2^ = 0.96), strongly suggest these values are likely accurate representations of SbtR-DNA binding kinetics under our experimental conditions.

Not all DNA-binding proteins yield such high-quality data as SbtR binding to its consensus sequence. Thus, there are several potential pitfalls regarding the use of this technique. Firstly, the buffer used throughout the protocol may have to be catered to your protein of interest. For example, if your protein has a high affinity to DNA through electrostatic interactions, you may need to decrease the concentration of nonionic detergent and increase the concentration of monovalent cations. These conditions are best assayed with known DNA-binding sequences and random DNA sequences. Additionally, at high protein concentrations, we usually observe a maximum shift of 0.5–1 nm. Values much lower than this range may be difficult to ascertain accurate kinetic values. If this is the case, one may try loading more DNA to the biosensor. Furthermore, the Octet system may produce artificially large shifts at the beginning of the experiment, likely due to the initial wetting of the biosensor tip. This should not affect experimental results, as each sample will be normalized to the beginning of the Association step; however, this initial shift can be avoided by pre-wetting the biosensor tips. Finally, not all proteins bind DNA through simple one-phase association and dissociation. Ligand-depletion, additional nonspecific binding sites, and multiphase association/dissociation may need to be taken under consideration. These alternatives may be explored using GraphPad Prism software [12]. However, it may be necessary to investigate association and dissociation steps independently.

## Supporting information

S1 File Step-by-step protocol, also available on protocols.io. (PDF)
