# Supplementary material for: Biolayer interferometry for DNA-protein interactions
Source: PLoS One. 2022 Feb 2;17(2):e0263322. doi: 10.1371/journal.pone.0263322 (PMC8809612; doi:10.1371/journal.pone.0263322)
Supplement: S1 File — (PDF) [file pone.0263322.s001.pdf]

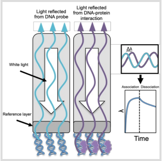

Biolayer Interferometry for DNA-Protein Interactions

John K Barrows[1], Michael Van Dyke[1]

[1]Department of Chemistry and Biochemistry, Kennesaw State University

1 Works for me

John Barrows
Department of Chemistry and Biochemistry, Kennesaw State Uni...

ABSTRACT

Biolayer interferometry (BLI) is a widely utilized technique for determining the interaction dynamics between macromolecules. Most BLI instruments, such as the Octet RED96e used throughout this protocol, are completely automated and detect changes in the interference pattern of white light reflected off a biosensor tip. Biosensors are initially loaded with a stationary macromolecule, then introduced into a solution containing macromolecules of interest. Binding to the stationary molecules creates a change in optical wavelength that is recorded by the instrument in real-time. The majority of published BLI experiments assess protein-protein (such as antibody-substrate kinetics) or protein-small molecule (such as drug discovery) interactions. However, a less-appreciated assay for BLI analysis is DNA-protein interactions. In our laboratory, we have shown the practicality of using biotinylated-DNA probes to determine the binding kinetics of transcription factors to specific DNA sequences. The following protocol describes these steps, including the generation of biotinylated-DNA probes, the execution of the BLI experiment, and data analysis through GraphPad Prism.

PROTOCOL INFO

John K Barrows, Michael Van Dyke . Biolayer Interferometry for DNA-Protein Interactions. **protocols.io**
https://protocols.io/view/biolayer-interferometry-for-dna-protein-interactio-bx9spr6e

FUNDERS ACKNOWLEDGEMENT

National Science Foundation
Grant ID: 2041202

KEYWORDS

biolayer interferometry, BLI, DNA-protein interaction

CREATED

Sep 15, 2021

LAST MODIFIED

Sep 23, 2021

PROTOCOL INTEGER ID

53266

MATERIALS TEXT

- New England Biolab *Taq* DNA Polymerase with Standard *Taq* Buffer (M0273)
- biotinylated DNA primer for PCR
- flat-bottom, natural, Greiner Bio 96-well microplate
- streptavidin Dip and Read^{TM} Biosensors (Forte Bio)
- Octet RED96e 8-Channel System
- GraphPad Prism software

PCR synthesis of biotinylated DNA probes 2h 15m

1 Our laboratory uses DNA probes derived from the ST2R24 selection template (sequence details can be found at: https://doi.org/10.1371/journal.pone.0159408) to identify the preferred binding sequence of thermophilic transcription factors. The design of related fluorophore-labeled modular selection templates and their synthesis has been described (https://dx.doi.org/10.17504/protocols.io.wfjfbkn). These DNAs are approximately 50-70 bp in length

and have been used extensively for BLI analysis.

2 Following manufacturer's guidelines (https://doi.org/10.17504/protocols.io.ch7t9m), assemble a 50 µL PCR reaction containing: 1× NEB Standard Taq Reaction Buffer, 200 µM dNTPs, 100 nM 5' biotin-labeled primer BIO_ST2R and 100 nM unlabeled primer ST2L, 4 ng ST2-derived template DNA, and 1.25 units Taq DNA polymerase in a 0.2 mL thin-wall PCR tube on ice. Mix thoroughly by pipetting. 15m

The biotinylated BIO_ST2R primer was purchased from Integrated DNA Technologies and contains a Biotin_dT at the 5' end. More information about biotin-conjugated nucleotides from Integrated DNA Technologies can be found at: https://www.idtdna.com/pages/education/decoded/article/which-biotin-modification-to-use-.

For an example experiment used throughout this protocol, we will perform BLI with the consensus binding sequence our laboratory identified for the *Thermus thermophilus* transcriptional regulation SbtR (Van Dyke MW et al. Identification of Preferred DNA-Binding Sites for the Thermus thermophilus Transcriptional Regulator SbtR by the Combinatorial Approach REPSA. PLoS One. 2016 Jul 18;11(7):e0159408. DOI: 10.1371/journal.pone.0159408. PMID: 27428627; PMCID: PMC4948773.). The ST2-derived template DNA used for our PCR contains the published consensus sequence, and the resulting PCR product will be further denoted as biotinylated-WT_SbtR.

3 Place PCR reactions in a thermal cycler (Bio-Rad C1000 Touch™). Design a program using a heated lid (105°C) for the following steps: (1) 95 °C, 2:00 min, (2) 95 °C, 0:30 min, (3) 54 °C, 0.30 min, (4) 68 °C, 1:00 min, (5) Go to Step 2, 19×, (6) 68 °C, 2:00 min, and (7) 4 °C, ∞. 1h

4 We suggest qualitatively analyzing PCR reaction products by native PAGE and ethidium bromide staining to ensure the formation of a single, double-stranded DNA PCR product. 1h

96-well plate set-up 20m

5 Our laboratory uses flat-bottom, natural, Greiner Bio 96-well microplates for our BLI experiments. During the automated BLI experiment, sensors are stationary within a single row and move between different columns. Therefore, each column will be filled with different solutions containing either DNA, protein, or buffer. For our reactions, we commonly use the buffer, BLI-100 (20 mM Tris, pH 7.5; 100 mM NaCl, 1 mM EDTA, and 0.05% Tween-20).

An example workspace is presented in **Figure 1**.

It is important to note that changes in the NaCl and Tween-20 concentrations can dramatically affect the binding and specificity of certain proteins based on electrostatic interactions. Titrating these reagents with a positive and negative control DNA probe is strongly recommended.

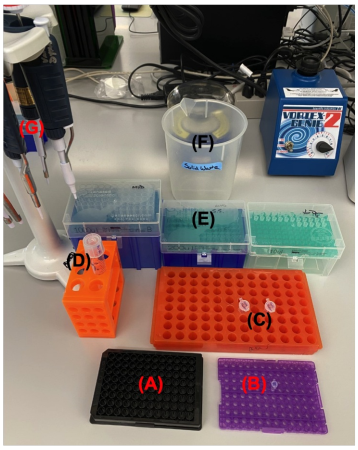

Figure 1: Workspace for BLI plate set up. (**A**) Black Greiner Bio 96-well microplate, flat bottom. (**B**) Biotinylated-WT_SbtR DNA generated by PCR as described in Steps 1-4. (**C**) DNA and protein dilutions in BLI-100 buffer. (**D**) BLI-100 buffer. (**E**) Micropipette tips. (**F**) Waste container. (**G**) Micropipettes.

Create a solution of BLI-100. Each well receives 200 µL, so we recommend preparing extra BLI-100 (250 µL per well) to 10m

6 account for minor pipetting deviations. For our example experiment in this protocol, we will be using Rows A-D and columns 1-4 (32 wells total, see **Figure 2**). Therefore, we suggest making 4 mL of BLI-100. Add 200 µL of BLI-100 to each well in Columns 2, 3, and 4. Columns 2 and 4 will be used for washes during the automated BLI experiment, and Column 3 will be used to dilute our protein of interest (discussed in step 8). All dilutions and preparation can be performed at room temperature.

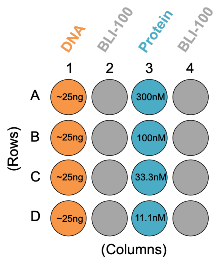

Figure 2: 96-well plate set-up for example experiment. Each well contains a 200 µL volume, and both DNA and protein dilutions are done with BLI-100 buffer.

7 Create a solution of biotinylated-DNA probes and BLI-100. We add ~25 ng biotionylated-DNA (~6 µL of our PCR reaction; steps 1-4) to 200 µL BLI-100 for each well. You should make a master mix of DNA/BLI-100 if you plan on using the same DNA template for multiple sensors. 2m

For our example, we will create a master mix containing 1 mL BLI-100 and 125 ng biotinylated-WT_SbtR, then aliquot 200 µL of this master mix to each well in Column 1.

The amount of DNA we use in each reaction does not saturate the streptavidin-coated biosensor; however, we have observed little differences in results when increasing the amount of DNA in each well. Conversely, significantly reducing the amount of DNA can affect loading efficiencies on the biosensor and lead to inconsistent results.

8 Create a solution of protein and BLI-100. We use several protein concentrations for our BLI experiments, and optimum amounts are often determined by first performing a wide range of concentrations. Protein stocks are initially diluted with BLI-100, then this stock is used to create 3-fold dilutions in Column 3 of the 96-well plate. To do this, 100 µL of the protein/BLI-100 mixture is added to well A3 (which already contains 200 µL BLI-100) and mixed by repeated pipetting. Then, 100 µL from well A3 is transferred to well B3 and mixed. This is continued to well D3, where 100 µL is removed from the well after mixing to retain a final volume of 200 µL. In our example, we will create a 900 nM SbtR/BLI-100 stock. Then, following the dilutions discussed above, the final concentrations of our wells will be: A3 - 300 nM; B3 - 100 nM; C3 - 33.3 nM; D3 - 11.1 nM. 5m

Program set up and execution of BLI experiment 1h 10m

9 Place your 96-well plate firmly within the Forte Bio's Octet RED96e system. Place the Streptavidin Dip and Read^{TM} Biosensors (FortéBio) in the appropriate position next to your plate. The entire tray that these biosensors are packaged in fits into the Octet RED96e. An example of a correctly placed plate and biosensor tray is presented in **Figure 3**. 2m

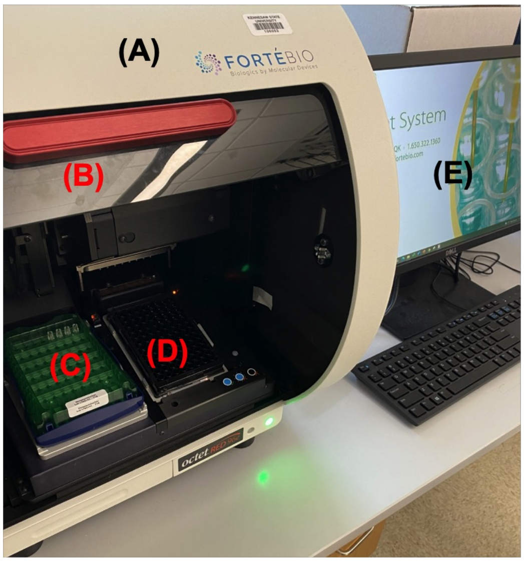

Figure 3: Octet RED96e Set Up. (**A**) FortéBio Octet RED96e. (**B**) Sliding glass door. (**C**) Biosensor tray containing four biosensor tips that will be used for the experiment. (**D**) 96-well plate holder containing a pre-loaded plate. (**E**) Associated computer for program set up and data curation.

10 Close the sliding door to the Octet RED96e and open the Data Acquisition 12.0 application on an associated computer. By opening this application, the Octet RED96e system will begin a self initialization check, which takes ~30 seconds. 2m

11 On the Experiment Wizard page, click on New Kinetics Experiment --> Basic Kinetics --> Blank Experiment. This will open up a page with 5 tabs: 1. Plate Definition; 2. Assay Definition; 3. Sensor Alignment; 4. Review Experiment; and 5. Run Experiment. 10m

11.1 **1. Plate Definition**
On the 96-well plate diagram on the left side of the window, highlight the wells that will be used in the experiment. Doing so will add the selected wells to the Plate Table shown on the right. Each highlighted well is initially labeled as "Sample"; however, this will need to be changed for your DNA and buffer-containing samples. For our example experiment, we need to highlight the wells in Columns 1 - 4, from Row A - D. We will then change the "Type" for wells A1 - A4 to "Load", as these wells contain our biotinylated-DNA probes. The "Type" for wells B1 - B4 and D1 - D4 will be changed to "Buffer", as these only contain BLI-100 buffer. Wells C1 - C4 should already be labeled as "Sample", which is correct since these wells contain dilution of our protein of interest, SbtR. Additional information, such as sample identification and molar concentration, can also be added to the Plate Table.

11.2 **2. Assay Definition**
We will now manually add steps to our assay in the Step Data List. A typical run consists of a sensor check, in which the biosensors are incubated in BLI-100 buffer. Then a Loading step, where the biosensors are transferred to a DNA-containing mixture. This is followed by a Baseline step, where the biosensors are transferred back to BLI-100 buffer. Next, we perform an Association step, in which the biosensors are moved to a protein-containing mixture. Finally, the biosensors are moved to a fresh BLI-100 buffer for a Dissociation step. This typical run-through for our laboratory is presented in the following table:

Name	Time	Shake Speed	Type	Threshold
Sensor Check	500	1000	Baseline	[unchecked]
Loading	900	1000	Loading	[unchecked]
Baseline	300	1000	Baseline	[unchecked]
Association	500	1000	Association	[unchecked]
Dissociation	900	1000	Dissociation	[unchecked]

Once the Step Data List is finished, click the grey box to the left of the "Sensor Check" step. A black arrow should appear. Next, highlight Column 2 in the 96-well diagram on the left side of the window. The easiest way to highlight an entire column is by clicking the blue-colored number at the top of the column. Once highlighted, click "New Assay" under the Assay Steps List. You should see a new row appear in the Assay Steps List table. To add the remaining steps within the same assay, click "Replicate" and select "Append to current assay". Adding steps in this way will keep the same biosensor between columns. Repeat this three more times to have a total of five steps in the Assay Steps List. The four replicated steps need to be edited to match our experimental design. Edit the Assay Steps List as shown in the table below:

Assay	No.	Sample	Step Name	Step Type	Sensor Type	Assay Time
1	1	2	Sensor Check	Baseline	SA (Streptavidin)	
1	2	1	Loading	Loading	SA (Streptavidin)	
1	3	2	Baseline	Baseline	SA (Streptavidin)	
1	4	3	Association	Association	SA (Streptavidin)	
1	5	4	Dissociation	Dissociation	SA (Streptavidin)	0:53:04

11.3 **3. Sensor Alignment**

This tab shows where the biosensors will be picked up from their tray. The biosensors for our example experiment will be picked up from the biosensor tray in the slots corresponding to wells A1 - A4. Double-check that biosensor tips are placed in the correct positions in the biosensor tray before moving to the next tab.

11.4 **4. Review Experiment**

Use the black arrows in the top right corner to review each step of your experimental design. The walk-through of our example experiment is presented in **Figure 4** below.

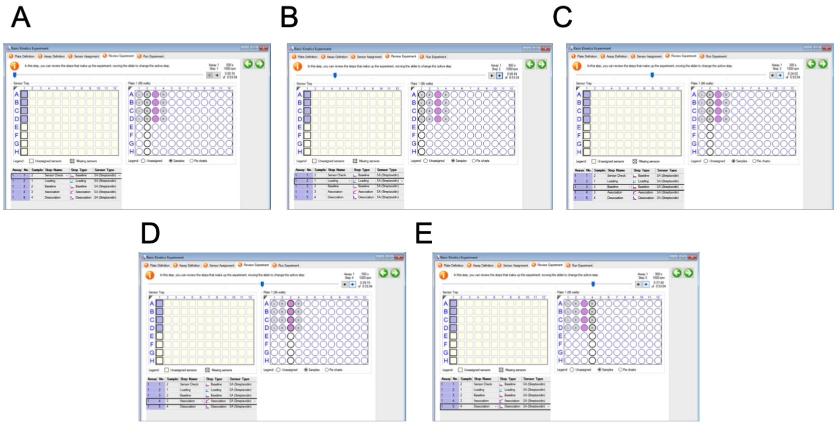

Figure 4: Experiment Review. (**A**) Step 1: Sensor Check in Column 2 (Buffer). (**B**) Step 2: Loading in Column 1 (DNA). (**C**) Step 3: Baseline in Column 2 (Buffer). (**D**) Step 4: Association in Column 3 (Protein). (**E**) Step 5: Dissociation in Column 5 (Buffer).

11.5 **5. Run Experiment** 1h

Save your run in a specified folder. We use the default run settings for our reactions; however, these can be adjusted as needed. Press the green "GO" button at the top right corner of the screen to start the experiment. Experiment runtime charts should open automatically to allow tracking of the

experiment in real-time. The run time of our example experiment is ~54 minutes.

Kinetics analysis using GraphPad Prism 20m

12 Open FortéBio Data Analysis 12.0. Load the saved folder containing your experiment by clicking File --> Load a Folder. Once loaded, you will be presented with a window containing three tabs at the top left corner: 1. Data Selection; 2. Processing; 3. Analysis. 1m

We routinely use GraphPad to analyze our BLI data, which will be discussed extensively below. However, the Data Analysis application can also be used for kinetic calculations. More information on how to use the Data Analysis software can be found by clicking "Help" --> "Data Analysis User Guide".

13 Click on the 2.Processing tab. Export raw data by clicking "Save Raw Data" on the left side of the window. This will export a file entitled "RawData0.xls" in your folder of choice. This excel spreadsheet contains the time (in seconds) and shift (in nanometers) for each row used in the experiment. 1m

14 To analyze association and dissociation rates, we will want to create a new table containing only the shift values for the Association and Dissociation steps of our experiment. In our example experiment, the Association step occurred at 1700 seconds. So, create a new table containing the shift values at time = 1700 seconds and beyond. Within each respective column, subtract all values by the shift value at 1700 seconds. Therefore each column should start with a shift equal to zero nanometers. Label each column with the corresponding protein concentration used. For simplicity, create a column for Time starting at 0. The Octet RED96e takes readings every 0.2 seconds, so your new Time column should increase by 0.2 increments for each shift reading. These data are now ready to be imported into GraphPad Prism for kinetics analysis. 5m

**Although our analysis primarily uses the Association and Dissociation data, it is important to look at the Loading data (which can be visualized in the Data Analysis 12.0 application or in the Data Acquisition 12.0 runtime charts). The binding of biotinylated-DNA probes to streptavidin-coated biosensors causes a small, but significant change in wavelength. It is important to check that this shift occurs and its amplitude is similar between wells with equivalent amounts of DNA.

15 Open GraphPad Prism (the version used in this example is 9.2.0 (332)). Create a new XY table. Under "Options" select "Numbers" for X and "Enter and pot a single Y value for each point" for Y. Copy and paste the Association and Dissociation values from the modified excel table in step 14 into the GraphPad XY table. Time (starting at 0) should be the X variable and the Y variables should be the shifts (in nanometers) for each experimental row. Each Y variable should be titled with the corresponding protein concentration (in nanomolar) used, as shown in **Figure 5** below. 5m

Table format: XY		X	Group A	Group B	Group C	Group D
		Time (s)	300	100	33.3	11.1
		X	Y	Y	Y	Y
1	Title	0.0	0.0000000	0.0000000	0.0000000	0.0000000
2	Title	0.2	0.0082046	0.0034273	0.0034638	-0.0060173
3	Title	0.4	0.0153885	0.0044048	0.0023323	-0.0012889
4	Title	0.6	0.0143063	0.0117822	0.0016289	0.0025189
5	Title	0.8	0.0203404	0.0070764	0.0028781	0.0018587
6	Title	1.0	0.0255846	0.0104954	0.0063437	0.0001032
7	Title	1.2	0.0339625	0.0138020	0.0099702	0.0021551
8	Title	1.4	0.0380688	0.0133585	0.0073647	0.0035325
9	Title	1.6	0.0516536	0.0161858	0.0081671	0.0000307
10	Title	1.8	0.0551950	0.0219118	0.0131190	0.0016671
11	Title	2.0	0.0525629	0.0189544	0.0085030	0.0068877
12	Title	2.2	0.0618305	0.0240622	0.0106831	0.0058977
13	Title	2.4	0.0634582	0.0251801	0.0153966	0.0010293
14	Title	2.6	0.0710557	0.0226104	0.0131105	0.0037683
15	Title	2.8	0.0777576	0.0314999	0.0115847	0.0064131
16	Title	3.0	0.0833756	0.0268582	0.0124023	0.0042543
17	Title	3.2	0.0887640	0.0314464	0.0127547	0.0083338
18	Title	3.4	0.0939150	0.0317114	0.0171468	0.0060751
[illegible]	[illegible]	[illegible]	[illegible]	[illegible]	[illegible]	[illegible]

Figure 5: Example of imported data in GraphPad Prism. The X variable is time in seconds, where time = 0 at the start of the Association step. The Y variables are titled based on the concentration of SbtR protein used in nanomolar units.

16 To analyze the data, click "Analyze" under the Analysis tab. You will be presented with a new window that asks which analysis you desire. For association and dissociation rates, we will select "Nonlinear regression (curve fit)" under the "XY analyses" section. Press "OK". 1m

17 A new window will appear entitled "Parameters: Nonlinear Regression". Under the first tab, "Model", select "Binding - 2m

Kinetics" --> "Association then dissociation". Then click on the "Constrain" tab. We will make several changes to these parameters.

- Change HotNM to "Data set constant (from column title)". This associates protein concentration (in nM) with each column title.
- Change "Kon" and "Koff" to "Shared value for all data sets". This produces global Kon and Koff rates.
- Change "Time0" to "Constant equal to" with a "Value" equal to the time in which dissociation starts. If you changed the Time in Step 14 to begin Association at Time = 0 seconds, then this value will be 500.

Press "OK" to begin analysis.

18 The results of the analysis will be presented in a table and include binding kinetics, confidence intervals, and goodness of fit parameters. The results can also be presented in graphical form under the "Graphs" tab. The results of our example experiment are presented in **Table 1** and **Figure 6** below. 2m

Kon (min-1*M-1)	Koff (min-1)	Kd (nM)	R-squared
282500	0.000234	0.827	0.96

Table 1: Association and dissociation rates for SbtR binding to its consensus sequence.

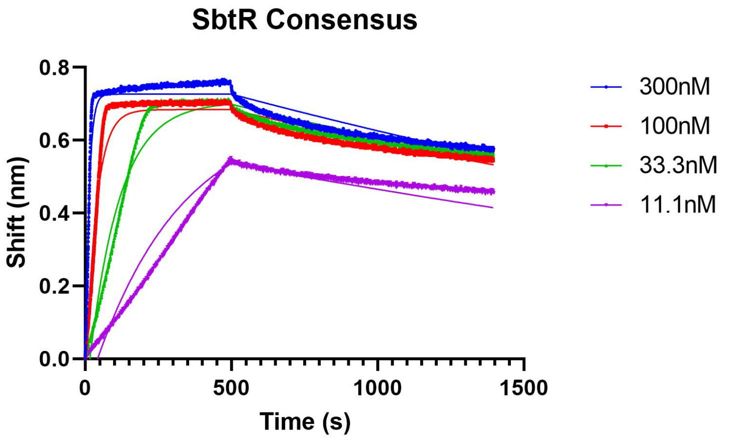

Figure 6: Graphical representation of BLI results showing binding of the thermophilic protein, SbtR, to its consensus DNA-binding sequence. Closed circles indicate individual data points and solid lines represent the nonlinear regression line of best fit.
